# Accuracy Evaluation of Additively and Subtractively Fabricated Palatal Plate Orthodontic Appliances for Newborns and Infants–An In Vitro Study

**DOI:** 10.3390/ma14154103

**Published:** 2021-07-23

**Authors:** Maite Aretxabaleta, Alexey Unkovskiy, Bernd Koos, Sebastian Spintzyk, Alexander B. Xepapadeas

**Affiliations:** 1 Department of Orthodontics in the University Centre of Dentistry, Oral Medicine and Maxillofacial Surgery within the University Hospital Tübingen, Osianderstr. 2-8, 72076 Tübingen, Germany; bernd.koos@med.uni-tuebingen.de (B.K.); alexander.xepapadeas@med.uni-tuebingen.de (A.B.X.); 2 Department of Prosthodontics, Geriatric Dentistry and Craniomandibular Disorders, Charité-Universitätsmedizin Berlin, Corporate Member of Freie Universität Berlin, Humboldt-Universität zu Berlin, Aßmannshauser Str. 4-6, 14197 Berlin, Germany; alexey.unkovskiy@charite.de; 3 Department of Dental Surgery, Sechenov First Moscow State Medical University, Bolshaya Pirogovskaya Street, 19c1, 119146 Moscow, Russia; 4 Section Medical Materials Science and Technology, University Hospital Tübingen, Osianderstr. 2-8, 72076 Tübingen, Germany; sebastian.spintzyk@med.uni-tuebingen.de

**Keywords:** digital workflow, SLA, DLP, trueness, precision, layer thickness, time efficiency, cleft palate plate, stimulation plate, Tübingen palatal plate

## Abstract

Different approaches for digital workflows have already been presented for their use in palatal plates for newborns and infants. However, there is no evidence on the accuracy of CAD/CAM manufactured orthodontic appliances for this kind of application. This study evaluates trueness and precision provided by different CAM technologies and materials for these appliances. Samples of a standard palatal stimulation plate were manufactured using stereolithography (SLA), direct light processing (DLP) and subtractive manufacturing (SM). The effect of material (for SM) and layer thickness (for DLP) were also investigated. Specimens were digitized with a laboratory scanner (D2000, 3Shape) and analyzed with a 3D inspection software (Geomagic Control X, 3D systems). For quantitative analysis, differences between 3D datasets were measured using root mean square (RMS) error values for trueness and precision. For qualitative analysis, color maps were generated to detect locations of deviations within each sample. SM showed higher trueness and precision than AM technologies. Reducing layer thickness in DLP did not significantly increase accuracy, but prolonged manufacturing time. All materials and technologies met the clinically acceptable range and are appropriate for their use. DLP with 100 µm layer thickness showed the highest efficiency, obtaining high trueness and precision within the lowest manufacturing time.

## 1. Introduction

**Figure 1:**
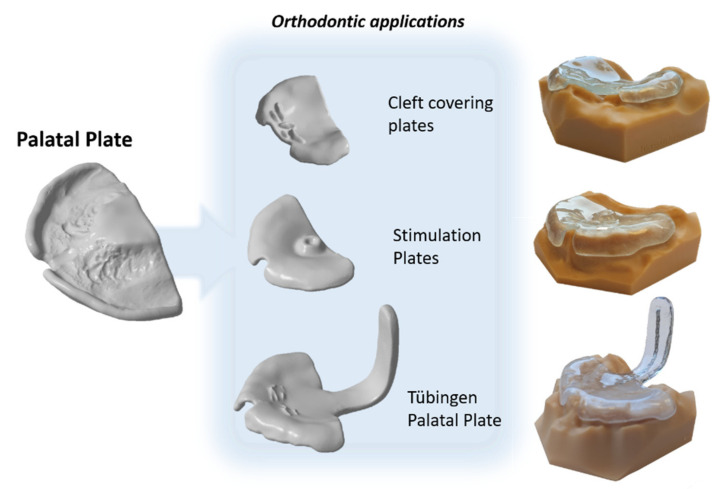
Digital palatal plate orthodontic applications: Cleft palatal plate, stimulation plate, Tübingen Palatal Plate (TPP). Digital files as well as the additively manufactured appliances on their respective model are exhibited.

Computer-aided design (CAD) and computer-aided manufacturing (CAM) have become relevant tools in the field of dentistry, steadily replacing conventional methods. Both subtractive (SM) and additive manufacturing (AM) are used in diverse dental applications, such as implant surgical guides [1], bite splints [2], complete dentures [3] and dental models for numerous applications (i.e., diagnostic models, orthodontic models) [4] and dental aligners [5]. Among the possible applications, there is a growing interest in the fabrication of orthodontic appliances for newborns and small infants, which are based on covering the edentulous maxilla. The applications are versatile (Figure 1): cleft palate plates, stimulation plates following the Castillo Morales concept (i.e., for Trisomy 21 patients) and the Tübingen Palatal Plate (TTP) for Robin Sequence (RS) patients. The cleft covering plate aims to cover the cleft and provide guidance for the alveolar segments to come closer together [6]. The stimulation plate aids in addressing speech impairment, feeding problems and breathing difficulties related to hypotonic tongue musculature [7,8,9,10]. The TPP aids in relieving the upper airway obstruction present in RS patients, by pushing the base of the tongue forward. When RS is accompanied by a cleft palate, the TPP is also responsible of covering the cleft area [11].

Orthodontic treatment of these patients starts with an impression (digital or conventional) of the upper jaw, as soon as possible after birth. For that purpose, intraoral scanning was found to be a safe and valid alternative to conventional alginate impressions [7,12], while removing the inherent risk of respiratory obstruction [13]. Moreover, alginate impressions must normally be carried out under close clinical supervision for these patients with craniofacial malformations, and most of the times might take place under general anaesthesia. Meanwhile, intraoral scanning can be performed in the orthodontic office. A learning curve when implementing scanning and software, as well as the associated costs are disadvantages of intraoral scanning [14]. In opposite, while being a safer procedure for the patient, the scanning also reduces patient discomfort and nausea sensation [15], while maintaining the accuracy of the conventional impression [16]. A digital workflow for direct appliance manufacturing has already been established for each palatal plate case [7,12]. In addition to making a physical model obsolete, it decreases hands-on time [11,15] and enables digital archiving [3,17].

Palatal plates are classified as MDR class IIa devices (European Medical Device Regulatory (MDR) 2017/745) [7,12]. In this category, splint materials are the standard. Conventionally, conventional cold polymerizing PMMA (such as, Orthocryl Clear, Dentaurum GmbH & Co. KG, Ispringen, Germany) are the state of the art [7,11,18]. Depending on the type of the palatal plate, though, both AM and SM can be considered. AM is based on fabrication a part layer by layer. These layers are formed by movement in the z axis. The size of each step defines the layer thickness, which is closely related to the accuracy of the part in the z-axis. In dentistry, as well as for the current application, vat polymerization AM technologies are of interest, specifically direct light processing (DLP) and stereolithography (SLA). Despite both technologies relying on curing photosensitive resin with UV light, the employed techniques differ. While DLP cures one complete layer at once, SLA creates the part by curing pixel by pixel in every layer (REF ADDMA TPP). The resin is contained in a transparent vat, into which a build platform is lowered. The UV light is projected onto the bottom of the resin container and the material is hereby selectively cured. The resolution of the printer in the x–y plane is defined by the resolution of the UV light projector (DLP) or the diameter of the laser (SLA) which are used for vat polymerization techniques. On the other hand, SM is based on the removal of material from a blank. The blank is fixed in the machine and spinning tools of different shapes and size gradually cut excess material from the blank until the desired shape is sculpted. DLP is particularly advantageous for simultaneous fabrication of multiple appliances [11]. Meanwhile, milled blanks are preferred in dentistry as they are less prone to suffer from shrinkage and deformation [19,20,21,22], as well as providing superior mechanical properties [18].

One of the factors influencing the success of palatal plate treatment is the retention of the orthodontic appliances to the edentulous maxilla [6]. Beyond safety and material performance, a high accuracy is imperative for palatal retention of the appliance [11,12]. Herein, manufacturing accuracy is a major concern. Based on ISO 5725-1:2019 for “Accuracy of measurement methods and results”, accuracy is a term addressing both trueness and precision. Trueness is defined as the closeness of agreement between the expectation of a measurement result and a true value. Precision instead, refers to the closeness of agreement between test results [23]. When it comes to stimulation plates, a difference in the fit was already observed, where AM-manufactured appliances fit better than SM fabricated appliances in patients [7]. The authors are unaware of any studies regarding both trueness and precision of CAD/CAM technologies for their application in this kind of orthodontic appliances.

The current study aimed to compare the trueness and precision of different AM, SM methods in combination with various materials and conventional method for the fabrication of palatal plates. The primary outcome of this study should be a solution to obtain the highest accuracy within the lowest manufacturing time. Different null hypotheses were defined for this study:

- The compared technologies do not differ in accuracy from the conventional solution.
- Layer thickness does not influence the accuracy for DLP samples.
- There is no significant difference in accuracy among the tested SM materials.

## 2. Materials and Methods

### 2.1. Sample Design and Manufacturing

**Figure 2:**
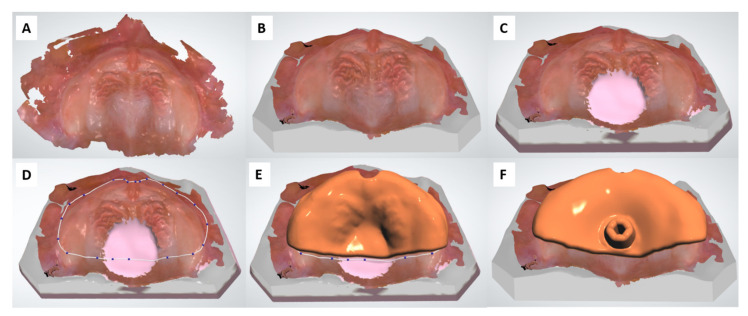
Plate design process in 3 Shape Appliance Designer and Dental Designer [7]. (**A**) Scan of the patient, (**B**) Model creation based on an intraoral scan, (**C**) Wax addition in regions of conflict in the model, (**D**) Definition of the plate area, (**E**) Construction of the plate, (**F**) Placement of the stimulation plate and smoothening of the plate.

A standardized palatal stimulation plate was designed in a complete digital workflow [7], based on an anonymized intraoral scan (Trios 3, 3Shape A/S, Copenhagen, Denmark), and selected as the sample for this study (Figure 2). The workflow comprises of creating a virtual model with Appliance designer (3Shape A/S). This is then transferred to Dental Designer (3Shape A/S) where digital wax is added in the palatal area and the palatal plate is designed virtually. In this case, a posterior stimulation element was also added (Figure 2). Samples were manufactured by AM (DLP, and SLA), SM and conventional methods. Sample size was selected according to established values from literature [3,4,17,19,24,25,26]. According to the pilot phase of the present study, a minimal difference in RMS value between all manufacturing groups was 0.005 mm. Given this fact, a sample size of 12 specimens for each manufacturing method was calculated with OpenEpi software [https://www.openepi.com/SampleSize/SSMean.htm (accessed on 15 of January 2021)] as sufficient to detect this difference using an alpha of 5% and 80% power. All samples were produced and post-processed by the same investigator (M.A).

**Figure 3:**
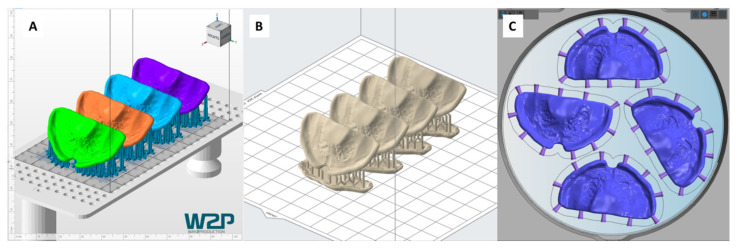
Sample positioning and supporting structures for the respective technology. (**A**) Positioning in Netfabb Premium 2019 for the Solflex 170 DLP device, (**B**) Positioning in Preform V3.01 for the Form 3B SLA device, (**C**) Positioning on DentalCAM for K5+ milling device.

For DLP, the material V-Print Splint (lot #2021055, VOCO GmbH & Co. KG, Cuxhaven, Germany) was printed using the Solflex 170 (W2P Engineering GmbH, Vienna, Austria). For job file preparation, Netfabb Premium 2019 (Autodesk, Mill Valley, CA, USA) was employed. The specimen was positioned in a clinically relevant position (65°) and supports were added (Figure 3). The stimulation plate was placed inclined, with the palatal side facing up to avoid possible surface defects caused by the attachment of support structures. For post-processing, manufacturer instructions were followed [27]. The washing procedure comprised of: 3 min 98% Isopropanol (IPA) cleaning in an ultrasonic (US) cleaner (Ulsonix Proclean, Expondo GmbH, Berlin, Germany), drying by compressed air, 2 min fresh IPA cleaning in US bath and ending with drying by compressed air. Before post-curing, support structures were removed. For the post-curing, a two-step curing process by Otoflash G171 (λ = 280–700 nm, NK-Optik, Baierbrunn, Germany) was carried out: 2000 flashes, turning over samples, 2 min cooling off (with opened lid) and further 2000 flashes.

SLA samples were manufactured with the material Dental LT Clear V1 (lot #XG394N02, Formlabs, Sommerville, MA, USA) in the Form 3B (Formlabs). PreForm V3.01 (Formlabs) was used to prepare the respective print file. The samples and their orientation were imported from the DLP printing file into PreForm V3.01 (Formlabs) as a .stl file (Figure 3), to ensure the exact same positioning for the different technologies. Once imported, supports were attached to the specimens, by the “auto generate supports” function. For post-processing, manufacturer instructions were followed [28]: washing for 20 min with 98% IPA in Formwash (Formlabs), drying by compressed air and post-curing for 20 min at 80 °C in Formcure (λ = 405 nm, Formlabs). Support structures were removed after complete polymerization. Each SLA and DLP job file (Figure 3) (comprising of four samples) was fabricated three times. For DLP, three different layer thicknesses were employed: 25, 50 and 100 µm. For each thickness, 12 samples were produced. In case of SLA, only 12 samples with 100 µm were manufactured, as only this layer thickness is printable with this splint material.

SM samples were manufactured in a five-axis dry milling machine (K5+ Milling machine, vhf camfacture AG, Ammerbuch, Germany). Two materials in blanks of 98.5 mm diameter and 25 mm thickness were considered: milled Polymethylmethacrylate (PMMA); Yamahachi PMMA (lot #MF42, Yamahachi Dental MFG Co., Gamagori, Japan) and milled Polyether-etherketone (PEEK); Smile PEEK (lot #01147, Pressing Dental S.r.l., Falciano, Republica di San Marino). Dental CAM and Dental CNC (vhf camfacture AG) software were employed for nesting, where the milling strategy “Denture” and optimal quality were selected. The functions “detection of cavities and drillings” were disabled. Four plates were manufactured per blank and support structures were laterally attached to each, avoiding the palatal area (Figure 3). A total of three milling tools were used (vhf camfacture AG): P250-R2-40, P200-R2-40 and P100-R2-40. For each SM material, 12 sampled were produced.

**Figure 4:**
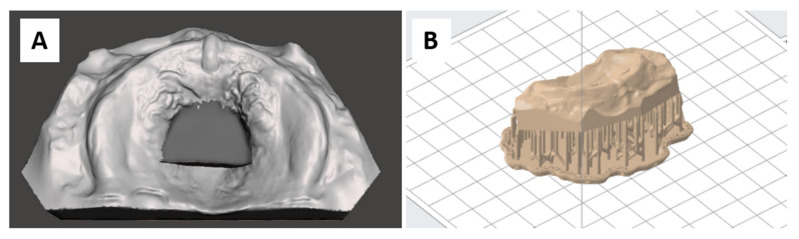
Model wax construction and printing preparation. (**A**): Wax placement in the palatal area of the model in Meshmixer 3.5. (**B**): Support placement and model positioning in Preform V3.01.

As a comparison to CAD/CAM manufactured plates, specimens of conventional material were produced on a physical model. For that, the virtually designed maxillary model (Figure 2) was additively manufactured, which resembles a semi-digital workflow [7]. As exporting the model with “wax” is not possible, the wax was added using a 3D mesh modelling software, Meshmixer 3.5. (Autodesk Inc., San Rafael, CA, USA). The digital model and palatal plate were imported into the software in STL format. The tool “Align to target” was used to automatically position the palatal plate on the model. Next, the palatal area of the plate was selected and extruded into the model to refill the area lacking of wax (Figure 4). After carefully smoothing the borders with the “robust smooth” command, the modified model was exported as an STL file. These were manufactured using Form 3B, PreForm V3.01., Model V2 (lot # 20190904RT, Formlabs) material and 100 µm layer thickness. One sample per print file was fabricated, placing it in the centre of the platform in a clinically relevant orientation, as shown in Figure 4. “Auto generate supports” function was employed for support placement. The same print file was manufactured 12 times. Models were post-processed following the manufacturer instructions [29]: 10 min washing with 98% IPA in Formwash, drying with compressed air and post-curing for 30 min at 60 °C in Formcure. Support structures were removed after complete polymerization.

Conventional plates were produced by an experienced dental technician. One plate was created per model. Separating medium (Sheraseparater, Lot #A2530, Shera Werkstofftechnologie GmbH & Co., Lemförde, Germany) based on wax was sprayed onto the model. This compound was left to dry for some seconds, before drying it completely by compressed air. In the following, a cold polymerizing PMMA called Orthocryl Clear (Dentaurum GmbH & Co. KG), which is comprised of liquid methyl methacrylate monomer (Lot #501141 A) and polymethylmethacrylate powder (Lot #480151 A) was applied on the model using the spray on technique defined by the manufacturer [30]. Samples were then polymerized in a pressure pot for 30 min at 40 °C and 3 bars. The plate was further post-processed following a conventional dental technician processing (removing excess material, smoothening, polishing). Two samples were manufactured per day, with the objective of reflecting a realistic scenario.

### 2.2. Comparison of Manufacturing Times and Material Cost

Manufacturing times for each group were measured, from the design phase until the appliance is prepared for its use in patients. The manufacturing time was analyed once per build job or four appliances (in the conventional case). Material cost per palatal plate was calculated for each technology and material.

### 2.3. Acquisition and Measurement of Data

Samples were scanned with the D2000 optical extraoral scanner (3Shape A/S, Copenhagen, Denmark), with an accuracy of 5 µm [31]. The scanner was calibrated before scanning each bunch. Each sample was positioned and fixed to the scanning platform with modelling clay, where the palatal area of the samples was positioned parallel to the camera module, as it is the region of interest. Specimens were steam cleaned, dusted off with compressed air and anti-glare spray (particle size: 2.8 µm) was applied (Helling 3D Laser Scanning Spray, Lot VAD-66520R06, Helling GmbH, Heidgraben, Germany). The product was sprayed twice, from different directions. Scanning data were stored in standard tessellation language (.STL) format and exported to Meshmixer 3.5. (Autodesk Inc.). Here, irrelevant areas for tissue adaptation (i.e., occlusal surfaces) were eliminated from the scan, as they could compromise the surface matching process, as well as cause delays and errors in the measurement software.

**Figure 5:**
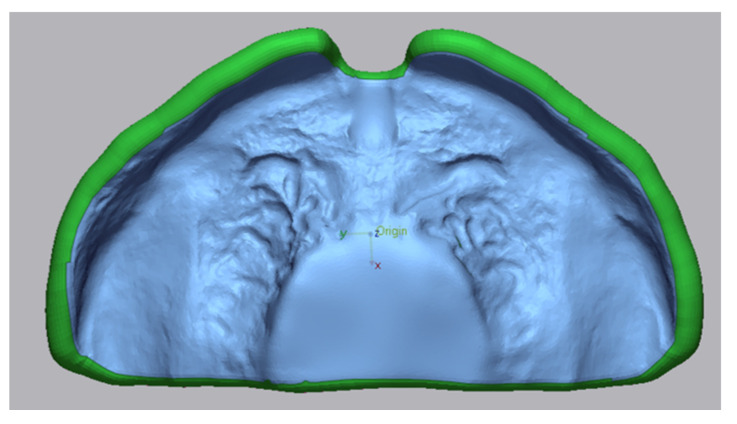
Selection of comparison area (blue) in the reference file for the superimposition process in Geomagic Control X 2020.

For obtaining trueness values, all the scanning files were compared to the original CAD file in a surface matching software (Geomagic Control X 2020, 3D systems, Rock Hill, SC, USA). The original palatal plate file was selected as the reference object in the program and an area of interest was selected in this file. For that, this area was trimmed in the file with the command “split”. This specific selected region is then used as the section to be compared in all cases (Figure 5). For precision measurement, samples within one group were compared. For that, the area of interested was trimmed and used as reference, following exactly the same selection criteria as for trueness (Figure 5).

The trimmed area was used to align the scanning file, by the commands “Initial Alignment” and “Best fit”. Afterwards, the same area was compared to the scanning file by “3D Compare”. Based on denture accuracy studies [3,17,24,25], a critical deviation of ± 300 µm (or acceptance range) and a nominal deviation of ±50 µm were defined for the colour maps. Surface deviation data was obtained by the software to report the degree of tissue adaptation, obtaining root mean squares (RMS) error estimates for trueness and precision. The objective of RMS is to assess the mean value of errors and the similarity of two N-dimensional vector sets under optimal superimposition [32]. A higher RMS is indicating a higher accuracy error. Therefore, a lower RMS value corresponds to higher trueness and precision. Moreover, complementary information of trueness is given: average deviations (positive and negative) as well as maximum deviations (positive and negative). By the “3D compare” command, a colour-coded map was also automatically generated for each superimposition analysis. A single investigator (M.A.) performed all scanning and superimposition processes for consistency purposes.

### 2.4. Statistical Analyses

Statistical analyses were performed using JMP 14 (SAS, Cary, NC, USA). To assess statistically significant differences among groups, the means of RMS were compared. First, all groups were subjected to normality and homogeneity of variance on a Shapiro–Wilk test. When the data was normally distributed, the means were compared by all pairs Tukey Kramer HSD. For non-normally distributed data, each pair Wilcoxon method was employed. A *p*-value of < 0.05 was considered statistically significant.

## 3. Results

### 3.1. Material and Technology Effect on Accuracy

Means and standard deviations for trueness RMS and precision RMS, as well as average and maximum mean for all tested technologies and materials are given in Table 1.

**Figure 6:**
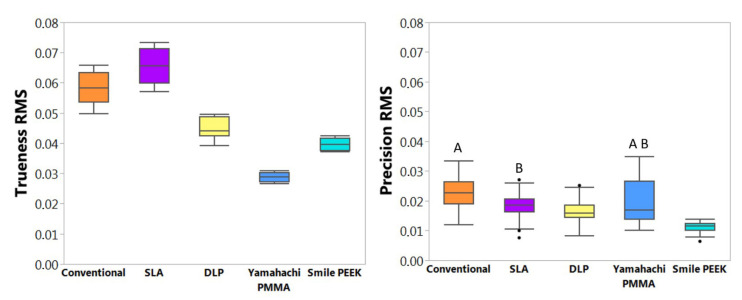
Mean trueness and precision root mean square (RMS) values (*n* = 12) for all groups. Trueness: normally distributed. Groups compared by all pairs Tukey–Kramer HSD analysis, all groups being significantly different (*p* < 0.05). Precision: non-normally distributed. Groups were compared by the Wilcoxon method (*p* < 0.05) and statistically comparable groups are denoted by letters. Lower RMS values correspond to higher trueness and precision quality.

The RMS data for trueness was normally distributed. All groups exhibited a significantly different trueness (*p* < 0.05) (Figure 6). The lowest RMS values were obtained by the milling technology, hence the highest trueness. Samples from Yamahachi PMMA (RMS = 0.029 ± 0.001) showed the lowest RMS values, as well as being significantly different to the other SM material, Smile PEEK (RMS = 0.040 ± 0.002). Among the AM materials, the DLP technology (RMS = 0.045 ± 0.003) showed the highest trueness, hence lowest RMS value. Except for SLA (RMS = 0.066 ± 0.006), all tested CAD/CAM technologies showed superior trueness in comparison to the conventionally manufactured group (RMS = 0.058 ± 0.005).

RMS data for precision was non-normally distributed. The highest RMS was obtained with conventional (RMS = 0.023 ± 0.005) and Yamahachi PMMA (RMS = 0.020 ± 0.008) groups, while they were statistically comparable (*p* = 0.5633) (Figure 6). Furthermore, a large standard deviation was observed for milled PMMA, and a statistically significantly higher (*p* < 0.05) RMS in comparison to milled PEEK was found. The lowest RMS value was obtained for Smile PEEK (RMS = 0.011 ± 0.002), offering the greatest precision. Among the AM technologies, DLP had superior precision (RMS = 0.016 ± 0.004) than SLA (RMS = 0.018 ± 0.004). SLA showed similar RMS values to Yamahachi PMMA (p = 0.0611) (Figure 6), while the rest of the groups were statistically significantly different (*p* < 0.05).

**Figure 7:**
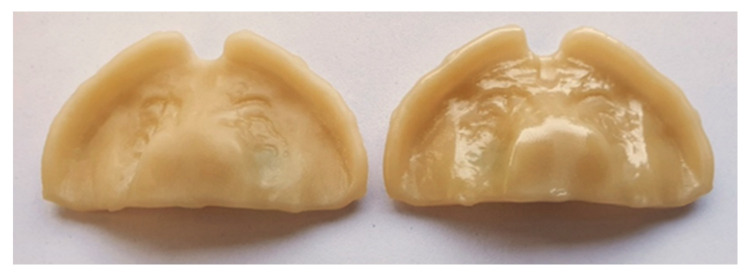
Difference of surface finishing between Yamahachi PMMA blanks after manufacturing. Left: manufacturing after PEEK blanks and used tools. Right: manufacturing with new tools.

The first Yamahachi PMMA blank (*n* = 4 samples) was manufactured after milling the PEEK blanks, with the same milling tools and showed a visibly lower surface quality (Figure 7). Although these tools had a large margin to the end of their designated lifetime, they were exchanged for the following two blanks, leading to an improved surface condition (Figure 7). Without considering the “defected” Yamahachi PMMA blank for the analysis (n= 8 samples remaining), the influence on trueness was minimal (RMS= 0.029 ± 0.001 to RMS= 0.028 ± 0.002). On the other hand, precision enhanced from RMS = 0.020 ± 0.008 to RMS = 0.014 ± 0.002. This value was still statistically significantly different to all groups but in this scenario, only PEEK had a higher precision.

An extensive accumulation of milling chips was observed for milling PEEK. In contrast to PMMA, the milling chip extraction system could not sufficiently remove PEEK residues during manufacturing as they adhered more to the inside of the machine.

**Figure 8:**
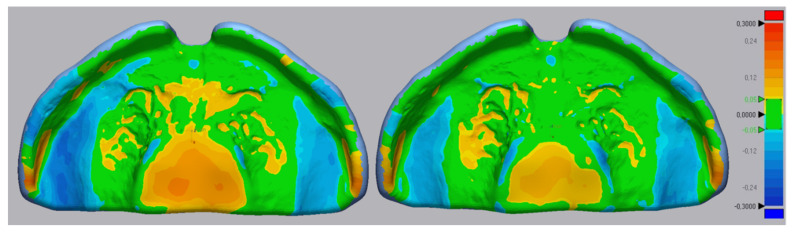
Exemplary files for the trueness color maps for the conventional samples. The color maps display a critical deviation of ±300 µm and a nominal deviation of ±50 µm (color range in mm).

Apart from the RMS values, colour maps provide a representation of the areas undergoing a deviation and the nature of these errors. In the conventional plates (Figure 8), there was a tendency of material accumulation in areas of the cleft, palatal rugae and posterior sides of the alveolar ridges. Comparatively, the top sides of the alveolar ridges showed a negative deviation. Despite these deviations, the conventional samples are within the defined range (±300 µm).

**Figure 9:**
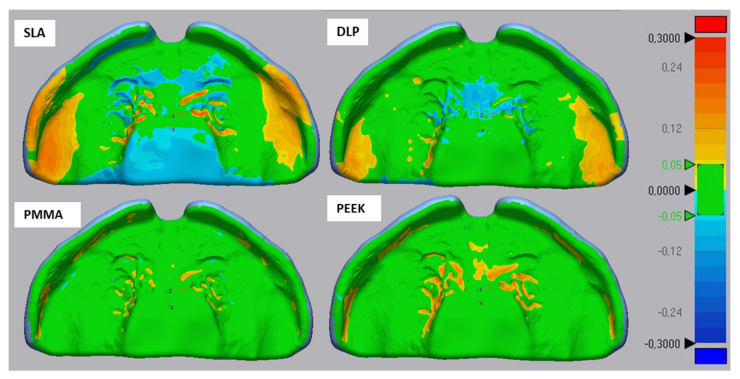
Exemplary trueness color maps of both AM technologies and materials (Dental LT Clear for SLA; V-Print Splint for DLP), as well as the two SM materials (Yamahachi PMMA and Smile PEEK) are displayed. The color maps display a critical deviation of ±300 µm and a nominal deviation of ±50 µm (color range in mm).

Exemplary trueness colour maps for AM and SM technologies are shown in Figure 9. When it comes to AM technologies, both DLP and SLA showed a tendency of material accumulation on the areas covering the alveolar ridges and the vestibule. This accumulation was more predominant in SLA. In DLP, the area of the palatal rugae presented a negative mismatch or material reduction to the reference CAD. In SLA samples, the mismatch in this area is comprised of both negative and positive deviations. The crevices corresponding to the palatal rugae showed an increase in material. The surrounding area to these crevices, as well as the area of the palate, had a reduction in material. Despite these errors, both AM technologies comply with the given acceptance range (±300 µm).

The SM materials presented mainly a positive mismatch in the regions of the palatal rugae and the side of the alveolar ridge (Figure 9). While both materials have trueness errors in similar sections, PEEK has a larger mismatch, also observed in the RMS data. Despite these deviations, both SM technologies comply with the proposed acceptance range.

### 3.2. Influence of Layer Thickness for DLP

**Figure 10:**
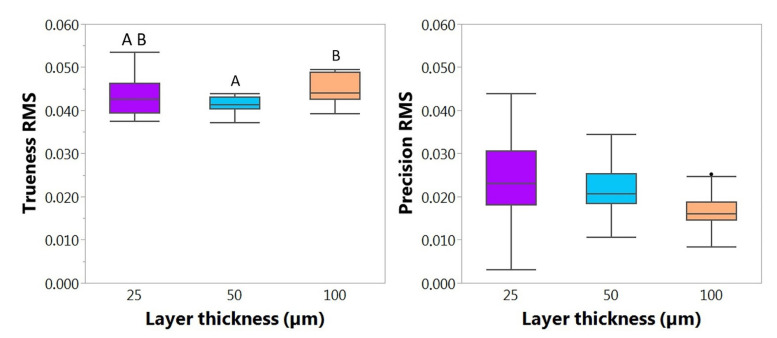
Mean trueness and precision root mean square (RMS) values (*n* = 12) comparison depending on layer thickness. All data is normally distributed. Groups compared by all pairs Tukey–Kramer HSD analysis (*p* < 0.05) and statistically comparable groups were denoted by letters. All groups from precision were statistically different (*p* < 0.05). Lower RMS values correspond to higher trueness and precision.

The RMS values of the 25, 50 and 100 µm groups followed a normal distribution for trueness. The 25 µm (RMS = 0.043 ± 0.005) group was statistically comparable in trueness to 50 µm (RMS = 0.041 ± 0.02; *p* = 0.4286) and 100 µm (RMS = 0.045 ± 0.003, *p* = 0.4286), while the latter groups were statistically significantly different (*p* < 0.05) (Figure 10; Table 2). The lowest layer thickness showed a larger standard deviation. Considering these data, the 50 µm layer thickness proved to have the lowest RMS as well as standard deviation and, therefore, also showed a greater trueness.

Regarding precision, the data is normally distributed, and all groups are significantly different (*p* < 0.05). Precision is significantly declining, and its standard deviation is increasing as the layer thickness decreases. Therefore, the highest layer thickness of 100 µm (RMS = 0.016 ± 0.004) showed the highest precision while the lowest was observed in 25 µm (RMS = 0.024 ± 0.008).

**Figure 11:**
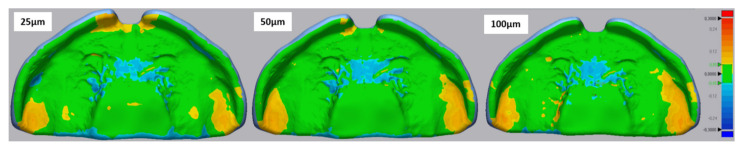
Exemplary files of trueness colour maps for the different DLP layer thicknesses (25 µm, 50 µm and 100 µm). The colour maps display a critical deviation of ± 300 µm and a nominal deviation of ±50 µm.

Regarding the colour maps (Figure 11), with increasing layer thickness, a slight material accumulation was observed in the alveolar ridge area. Comparatively, the positive deviation increases in the premaxilla area as layer thickness decreases. All samples showed a negative deviation in the area of the palatal rugae. All layer thicknesses showed trueness values within the defined clinically accepted range (±300 µm).

### 3.3. Comparison of Manufacturing Times and Material Costs

Information about the manufacturing times for the different technologies and materials is given in Table 3. The respective layer count is shown for AM. In DLP, printing time increases along with layer thickness (layer count).

In respect to AM technologies, despite having a similar layer count at 100 µm, SLA required more than double the time to print the same appliance. When reducing the layer thickness for DLP, longer manufacturing times were necessary. Milling had the longest manufacturing times, while having the shortest post-processing. Comparatively, post-processing time was longer for the AM technologies.

Regarding material cost (Table 3), the most inexpensive solution is the conventional material, followed by the AM materials. Milled blanks are considerably more expensive, where PEEK had the highest price.

## 4. Discussion

Despite no accuracy studies existing for palatal plates, the principle of function is similar to complete dentures, as the appliance bearing tissue is edentulous as well. Nonetheless due to the different scale and sizing of the appliances, as well as the different material used, the results from palatal plate accuracy might vary. Although there are accuracy research studies in splint materials [2], as well as for dentures [33], potential material combinations and technologies (conventional, AM, SM) have not yet been tested under uniform testing conditions for the given application.

### 4.1. Material and Technology Effect on Accuracy

All tested CAD/CAM samples had a statistically significant difference in accuracy to the conventional group and all groups met the clinically acceptable threshold of ±300 µm. Therefore, both statistical null hypotheses on that behalf had to be rejected. Considering the overall measurement and manufacturing procedure, the range in which the measured values are located might be close to the accumulated measurement accuracy of scanning device and matching software. Nevertheless, the following distinctive conclusions can be drawn from the results.

#### 4.1.1. Vat Polymerization Techniques

Considering AM technologies, significant differences in both trueness and precision were found (Figure 6). Both are vat polymerization techniques, but they employ different resin curing mechanisms. While DLP cures a complete layer at once, SLA creates the part by curing pixel by pixel in every layer [18]. DLP showed a significantly improved trueness and precision at 100 µm in comparison to SLA. Values found in literature for both SLA and DLP employed in denture and bite splints, comprised of higher RMS values than the ones observed in this study [2,3,19,24,25,33], hence lower accuracy. Some studies agreed with the superiority of accuracy of DLP in comparison to SLA found in this study [34], while other ones exhibit greater accuracy for SLA [33]. There is no consensus on which AM technology offers greater accuracy.

The result comparison between studies is challenging, due to the influence of extraoral scanner accuracies, materials and technologies, as well as accuracy evaluation methodology or protocol [19,26]. Comparing different materials is complex, as each has its own activation range, wavelength, intensity, different exposure times (depending on used printer) and curing depth [2,19]. Additionally, the manufactured geometries have an influence on the obtained accuracy values [34]. The palatal plates are smaller than other AM dentistry applications such as dentures and bite splints. Dimensionally larger and/or larger curved surfaces are more prone to the staircase effect than vertical surfaces, which leads to higher dimensional errors [33]. It might also be that when printing smaller samples, less mechanical forces need to be exerted for lifting the printing platform, which might have led to smaller surface deviations and deformations.

The effect of the build angle was not evaluated in this study. This is known to influence not only the accuracy but also the manufacturing time of vat polymerization techniques [2,3,11,33]. Vertical positioning, leads to an increase in layers, and thus, the sum of repeated errors or inaccuracies grows [2]. Considering that other studies comprised of larger geometries, they are more likely to be positioned vertically to fit onto the build platforms. In the contrary, a more horizontal positioning has the opposite effect on the layer count, while the samples can suffer from a higher overcuring effect. To improve the connection between layers, the polymerization extends the pre-set layer thickness, leading to an overcuring effect on the previous layer. Additionally, this effect might have a greater influence on transparent materials (as in this study), as the light might disperse more than in coloured materials [2], i.e., denture materials. Moreover, this phenomenon might lead to inhomogeneous polymerization that can be responsible for an uncalculated shrinkage behaviour [2]. This could be one of the reasons for the observed deviations in both AM technologies. While the same printing angle was employed in the both AM technologies considered in this study, the employed angle is one other factor that complicates the comparison to other studies. Other important design considerations that may influence the results, compared to other studies, comprise of aspects such as support placement and thickness, cupping errors influenced by orientation of the part and raft thickness.

Another factor that might have influenced the accuracy of vat polymerized parts are the post-processing protocols. They can influence different material characteristics, such as biocompatibility, mechanical properties and even accuracy [35]. During washing, the objective is to remove unreacted resin from the part’s surface. A US bath, as used for the DLP sample, is known to be more effective than Formwash which is stirring the cleaning solution around the part [36]. If remains of unreacted resin are present on the sample, this will be then post-cured, which could explain the higher positive deviation observed in the alveolar ridge for SLA samples in comparison to DLP (Figure 9).

When it comes to both AM technologies, factors such as layer thickness can be decisive for the accuracy. By decreasing layer thickness, the z-resolution increases, which might allow for a more detailed and improved surface [34]. Therefore, it is often assumed that accuracy improves as well. While some studies are in favour of this assumption [34], some suggest the opposite [4]. Additionally, changing the layer thickness influences manufacturing time, which is an important aspect to consider for the daily practice. The influence of layer thickness cannot be studied for Dental LT, as it is only printable in 100 µm. Even though lower layer thicknesses have proved to be beneficial in other accuracy studies for SLA [34], the manufacturing time would be excessive (Table 3) and not appropriate for the given application. Comparatively, DLP samples were fabricated in three different layer thicknesses, with the objective of finding the optimum ratio between sufficient accuracy and minimum manufacturing time. A statistically significant difference in trueness was found between 50 µm and 100 µm, where 50 µm was superior (Figure 6). As there is a significant change in the result of accuracy depending on the layer thickness, the null hypothesis was, therefore, rejected. 25 µm showed a higher standard deviation, while being comparable to both 50 µm and 100 µm in trueness, but statistically significantly different in precision. By decreasing layer thickness, the accuracy was partially improved: while obtaining an improved trueness with reduction of layer thickness to 50 µm, this value was worsened at 25 µm. Moreover, it was seen that the RMS precision values increased with the reduction of layer thickness, which indicates a reduction of reproducibility. When selecting a higher resolution in the z axis and hence, reducing the layer thickness, the number of printed layers linearly increases. This might be accountable for causing a higher chance of errors, artefacts and failures over the course of a print, and may lead to lower trueness as well as precision for DLP [4]. In addition to this, the manufacturing time for the same sample increases considerably (Table 3).

#### 4.1.2. Milling

Both milled materials exhibited a high trueness and precision, significantly higher than the other groups (*p* < 0.05). These results are in agreement with the values found in the literature, proving that milled appliances comprise of higher accuracy than AM ones [2] as well as conventional ones [17,20,22,37]. Moreover, both materials were significantly different to each other; therefore, the null hypothesis was rejected. A significantly higher trueness was found for Yamahachi PMMA (Figure 6). While having the same milling procedure, Smile PEEK is known to have an increased hardness and higher flexural strength [18], which can pose a difficulty when milling. This led to a visibly rougher surface finish for Smile PEEK and might be responsible for the reduced trueness value. Comparatively, Smile PEEK presented the highest precision. Concerning Yamahachi, worse precision results were obtained due to the first blank which was milled with the same tools used for the PEEK samples before (Figure 7). Even though the tools where within their indicated lifetime, the tools might have suffered from additional wear due to the mechanical properties of PEEK.

For both SM groups, positive deviations were observed in the palatal rugae and the alveolar ridge (Figure 9). In palatal rugae region, this positive deviation might be caused by the limited cutter radius size. The side of the alveolar ridge poses an additional challenge for the technology, as regions with undercuts are difficult to machine [21].

#### 4.1.3. Conventional

The conventional material exhibited a trueness superior to the SLA technology (Figure 6). The deviations observed in the colour map (Figure 8) as well as lower trueness could be a result of different factors.

This study was based on direct intraoral scanning of the patients [7,12]. Manufacturing a physical model to create the conventional samples could have introduced an error depending on the accuracy of this model (material, positioning, supporting structures) [38]. Additionally, adding the wax digitally to the model to recreate the correct geometry in the conventional plate may have also produced an error in the palatal area. As a further influential factor, the model separating medium might slight down the slopes and crevices of the model and, therefore, accumulate in lower areas. This influence on accuracy remains to be investigated as no clear pattern could be identified in this study.

For producing the plate, the spray-on technique is recommended by the manufacturer [30]. The exact monomer to polymer ratio can vary due to the manual process, which leads to different shrinkage during polymerization and can result in a tighter or looser fit of the appliance [11,19]. This shrinkage might have contributed to the deviations observed in the colour map [19,22]. Apart from affecting accuracy, monomer content is known to influence the mechanical properties and the amount of unreacted residual material, among others [18]. Considering the obtained precision, the lowest values were observed for the conventional group, which is reflecting the manual process.

#### 4.1.4. Comparison Problems and Influence of Background Noise throughout the Measuring Workflow

Despite all precautions to create a reproducible manufacturing as well as measuring protocol, added minimal error or background noise through the process can be expected. This error is unmeasurable with the current technologies. In a worst-case scenario, the background noise might be larger than the measured differences between technologies. It is important to note, that the obtained accuracy values in this study were larger than differences between groups. This might lead to a misinterpretation of the statistical data.

There is little knowledge on the error that is propagating throughout the digital workflow. Lee et al. described complications to know exactly in which step of the manufacturing process errors are occurring [19]. The employed scanning powder has a particle size of 2.8 µm, which is below the accuracy of the scanner (5 µm). In vitro studies on the influence of scanning powder suggest there is no significantly decrease in scanning accuracy due to the small particle size [19]. Moreover, since the measured values are larger than the accuracy of the scanner, the measuring protocol appears to be adequate for comparing the accuracy differences between groups. Superimposition procedure is a well-established methodology in literature to assess the accuracy of appliances in vitro [2,3,17,19,24,25,33]. Despite this, no information exists on the calculation error (matching error) and relation of this data to in vivo results.

For manufacturing, there is a need to study the effect of the positioning of the parts on the printing platform [4,25,39], as it might influence the obtained accuracy. This was not considered in this study as the deviation values are so small, even when not considering the positioning, the clinically accepted range for this application was never exceeded.

### 4.2. Clinical Interpretation and Acceptance

**Figure 12:**
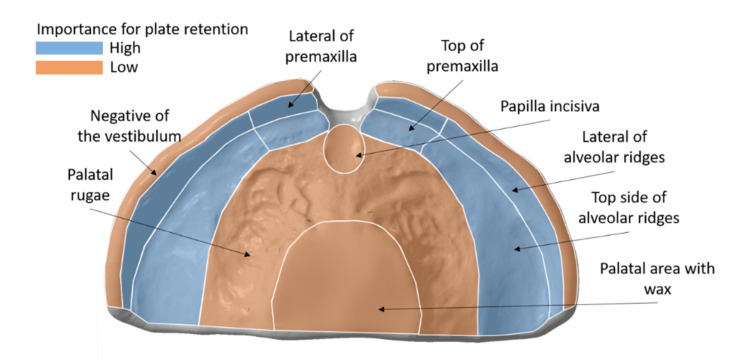
Schematic view of the palatal plate area in contact with tissue, its regions and their contribution to the appliance retention.

In palatal plates, the appliance bearing tissue portion is predominantly responsible for its retention (Figure 12). These areas consist of less resilient mucosa that relies on top of a bone structure and allows for controllable compression of the skin/mucosa. These areas comprise of: the premaxilla or primary maxilla and the area of the alveolar ridges. While the latter are necessary for a proper retention, the vestibule is expected to be inaccurate in the appliance, due to reflections of mucosa influencing the scan quality. Moreover, this area might be affected by the later post-processing steps of the appliance. Comparatively, areas such as the palatal rugae are formed by a thicker mucosa and no supporting structure underneath, where a larger compression margin exists. Moreover, it is prone to create more movement or sliding of the appliance. Therefore, in this area, the appliance cannot have a proper retention. Finally, the area covering the cleft or the palatal area that is covered in wax is not relevant when it comes to the retention.

While the objective of this study was to access the accuracy level, considering both trueness and precisions values, this information must be addressed from a clinical point of view. All studied groups complied with the proposed acceptance range for trueness of ±300 µm. Therefore, the null hypothesis was discarded, as all groups are clinically acceptable. From other side, precision is necessary to ensure that the same appliance can be manufactured with the same accuracy standards as a previous one. Moreover, precision values demonstrate the reproducibility and, therefore, the reliability of the employed devices. High precision values were obtained when fabricating the same file three times per technology, which is an indicator for manufacturing repeatability.

Considering this information as well as the colour maps, it is possible to assume the fitting in the maxillary model based on these in vitro results for the different tested groups. A positive deviation of the colour map can indicate mucosal compression or tissue impingement when placed in the patient, as an accumulation of material is observed. On the other hand, a negative deviation indicates a looser fit in the patient. Concerning the conventional plates, the cleft area, palatal rugae and posterior sides of alveolar ridges might suffer from compression, whereas the top sides of the alveolar ridges will sustain a looser fit. Plates produced by both AM technologies might induce compression in the alveolar ridges and the vestibule. In SLA the compression occurs over a larger area. DLP showed a looser fit in the palatal rugae area, while SLA showed a mixture of loose fit (in areas surrounding crevices) and compression areas (crevices). Reducing the layer thickness in DLP resulted in a reduction of the compression region seen at 100 µm in the alveolar ridges in combination with an increase in compression in the region of the premaxilla. SM plates showed areas in the side of the alveolar ridge as well as the palatal rugae crevices prone to tissue compression. As can be seen in Figure 12, the regions of the alveolar ridges and the premaxilla are the most important regions to provide a good retention of the appliances and, therefore, a higher accuracy is aimed for in these areas. Considering the colour maps, SM plates showed superior fitting or retention.

While all the technologies proved to have suitable accuracy for the clinical use, from the obtained results, different recommendations can be suggested. SM and SLA should be employed for applications were a greater surface finishing and higher mechanical properties are necessary. The TPP for example, requires a high flexural strength [11,18]. From the other side, DLP should be prioritized in cases where a faster manufacturing time and higher trueness and precision are necessary, for example prototyping of the TPP and manufacturing of cleft palatal plates. When deciding on the most appropriate material, besides material properties or accuracy, economic factors must be considered as well. Only calculating material costs can give a first impression while the overall costs which come along with the whole workflow must be calculated (i.e., machines, work force, tooling). Given that all groups showed superior accuracy quality, each facility should decide for the advantages that each technology offers and consider their available resources to make the appropriate decision.

### 4.3. Limitations

In the current study, different limitations can be mentioned. To start, the tissue surface adaptation of the appliance was only evaluated in vitro, considering that currently there is not enough knowledge of how these results correlate to the results in vivo [17]. Moreover, a protocol for reproducing different tissue compression does not exist yet. Apart from the accuracy when manufacturing, other considerations may play a role in the retention of the plates: intraoral scanning data, plate design process in the software and aspects such as surface roughness of the appliance and the effect of adhesive cream, which might be used in the clinical setting.

The materials were not analyzed under water storage, which may have an influence over the accuracy results obtained for conventional and AM materials, due to a higher degree of water uptake. Furthermore, the influence of other printing parameters as well as support placement was not studied. It is also important to mention that the current study did not evaluate all materials and devices available in the market and, hence, the results may vary.

## 5. Conclusions

Within the limitations of this in vitro study, the following conclusions can be drawn:

- All tested materials and technologies are within the clinically acceptable range and are appropriate for their use.
- A significant difference for the accuracy between two SM materials was found.
- SM offered superior trueness and accuracy than AM.
- DLP offered a superior trueness and precision values compared to SLA.
- All CAD/CAM groups showed superior trueness and precision to conventional manufacturing; except for SLA, which exhibited lower trueness but higher precision.
- DLP with 100 µm layer thickness showed the highest efficiency, obtaining high trueness and precision within the shortest manufacturing time.

## Figures and Tables

**Table 1:** Results from the superimposition process. Means and standard deviations are given for all groups for the following parameters: root mean square (RMS) of trueness, RMS of precision, positive and negative average deviation, as well as maximum and minimum deviations.

Measured Value		Conventional	AM-SLA	AM-DLP ^1^	SM-PEEK	SM-PMMA
Trueness (RMS, mm)	Mean	0.058	0.066	0.045	0.040	0.029
	SD	0.005	0.006	0.003	0.002	0.001
Precision (RMS, mm)	Mean	0.023	0.018	0.016	0.011	0.020
	SD	0.005	0.004	0.004	0.002	0.008
Average + (mm)	Mean	0.043	0.053	0.037	0.037	0.024
	SD	0.004	0.030	0.004	0.002	0.002
Average − (mm)	Mean	−0.081	−0.047	−0.035	−0.019	−0.016
	SD	0.120	0.008	0.003	0.002	0.002
Max + (mm)	Mean	0.200	0.264	0.168	0.171	0.123
	SD	0.071	0.053	0.017	0.009	0.006
Min − (mm)	Mean	−0.201	−0.153	−0.146	−0.076	−0.088
	SD	0.027	0.050	0.030	0.005	0.019

^1^ Only DLP and SLA groups with 100 µm layer height were considered.

**Table 2:** Values from the superimposition process for the different layer thicknesses. Means and standard deviations are given for the following parameters: root mean square (RMS) of trueness, RMS of precision, positive and negative average deviation, as well as maximum and minimum deviations.

Measured Value	DLP Layer Thickness			
		25 µm	50 µm	100 µm
Trueness (RMS)	Mean	0.043	0.041	0.045
	SD	0.005	0.002	0.003
Precision (RMS)	Mean	0.024	0.022	0.016
	SD	0.008	0.005	0.004
Average + (mm)	Mean	0.037	0.031	0.037
	SD	0.004	0.003	0.004
Average − (mm)	Mean	−0.035	−0.034	−0.035
	SD	0.003	0.002	0.003
Max + (mm)	Mean	0.148	0.146	0.168
	SD	0.023	0.022	0.017
Min − (mm)	Mean	−0.180	−0.160	−0.146
	SD	0.039	0.029	0.030

**Table 3:** Manufacturing time for producing four palatal plates at once for the different technologies and materials: conventional, stereolithography (SLA), direct light processing (DLP) and milling. Information on layer thickness and layer count are also provided for additive manufacturing (AM) technologies. Cost per part is shown for each material.

Technology	Material	Layer Thickness	Layer Count	Fabrication Time	Post-Processing Time	Total Time	Material Cost
Conventional	Orthocryl Clear	/	/	1 h 10 min	45 min	1 h 55 min	EUR 0.24
SLA	Dental LT Clear	100 µm	236	1 h 40 min	1 h 20 min	3 h	EUR 0.78
DLP	V-Print Splint	25 µm	914	1 h 55 min	27 min	2 h 22 min	EUR 0.77
		50 µm	457	1 h 1 min		1 h 28 min	
		100 µm	228	37 min		1 h 4 min	
Milling	Yamahachi PMMA	/	/	2 h 38 min	7 min	2 h 45 min	EUR 8.23
	Smile PEEK	/	/	3 h 18 min		3 h 25 min	EUR 39.13

## Data Availability

The data that support the findings of this study are available from the corresponding author, Maite Aretxabaleta, upon reasonable request.

## References

[1] Henprasert P., Dawson D.V., El-Kerdani T., Song X., Couso-Queiruga E., Holloway J.A. (2020). Comparison of the Accuracy of Implant Position Using Surgical Guides Fabricated by Additive and Subtractive Techniques. J. Prosthodont..

[2] Reymus M., Hickel R., Keßler A. (2020). Accuracy of CAD/CAM-fabricated bite splints: Milling vs 3D printing. Clin. Oral Investig..

[3] Jin M.C., Yoon H.I., Yeo I.S., Kim S.H., Han J.S. (2020). The effect of build angle on the tissue surface adaptation of maxillary and mandibular complete denture bases manufactured by digital light processing. J. Prosthet. Dent..

[4] Favero C.S., English J.D., Cozad B.E., Wirthlin J.O., Short M.M., Kasper F.K. (2017). Effect of print layer height and printer type on the accuracy of 3-dimensional printed orthodontic models. Am. J. Orthod. Dentofac. Orthop..

[5] Tartaglia G.M., Mapelli A., Maspero C., Santaniello T., Serafin M., Farronato M., Caprioglio A. (2021). Direct 3D Printing of Clear Orthodontic Aligners: Current State and Future Possibilities. Materials.

[6] Hotz M., Gnoinski W. (1976). Comprehensive care of cleft lip and palate children at Zurich University; A preliminary report. Am. J. Orthod..

[7] Xepapadeas A.B., Weise C., Frank K., Spintzyk S., Poets C.F., Wiechers C., Arand J., Koos B. (2020). Technical note on introducing a digital workflow for newborns with craniofacial anomalies based on intraoral scans—Part I: 3D printed and milled palatal stimulation plate for trisomy 21. BMC Oral Health.

[8] Castillo-Morales R., Crotti E., Avalle C., Limbrock G. (1982). Orofaziale regulation beim Down-syndrom durch gaumenplatte. Sozialpadiatrie.

[9] Castillo-Morales R., Brondo J., Hoyer H., Limbrock G. (1985). Die Behandlung von Kau-, Schluck-und Sprechstörungen bei behinderten Kindern mit der orofazialen Regulationstherapie nach Castillo-Morales: Aufgabe für Pädiater und Zahnarzt. Zahnärztl Mitt.

[10] Limbrock G., Hesse A., Hoyer H. (1987). Orofaziale Regulationstherapie nach Castillo-Morales bei Kindern mit zerebralen Läsionen. Fortschr. Kieferorthopädie.

[11] Aretxabaleta M., Xepapadeas A.B., Poets C.F., Koos B., Spintzyk S. (2021). Fracture Load of an Orthodontic Appliance for Robin Sequence Treatment in a Digital Workflow. Materials.

[12] Xepapadeas A.B., Weise C., Frank K., Spintzyk S., Poets C.F., Wiechers C., Arand J., Koos B. (2020). Technical note on introducing a digital workflow for newborns with craniofacial anomalies based on intraoral scans—Part II: 3D printed Tubingen palatal plate prototype for newborns with Robin sequence. BMC Oral Health.

[13] Chate R.A. (1995). A report on the hazards encountered when taking neonatal cleft palate impressions (1983–1992). Br. J. Orthod..

[14] Weise C., Frank K., Weise H., Reinert S., Koos B., Xepapadeas A.B. (2021). Intraoral scanning of neonates, infants, and small children with craniofacial disorders: Evaluation of feasibility, scanning duration and clinical experience. Eur. J. Orthod..

[15] Gallardo Y.R., Bohner L., Tortamano P., Pigozzo M.N., Lagana D.C., Sesma N. (2018). Patient outcomes and procedure working time for digital versus conventional impressions: A systematic review. J. Prosthet. Dent..

[16] Chebib N., Kalberer N., Srinivasan M., Maniewicz S., Perneger T., Muller F. (2019). Edentulous jaw impression techniques: An In Vivo comparison of trueness. J. Prosthet. Dent..

[17] Goodacre B.J., Goodacre C.J., Baba N.Z., Kattadiyil M.T. (2016). Comparison of denture base adaptation between CAD-CAM and conventional fabrication techniques. J. Prosthet. Dent..

[18] Aretxabaleta M., Xepapadeas A.B., Poets C.F., Koos B., Spintzyk S. (2021). Comparison of additive and subtractive CAD/CAM materials for their potential use as Tübingen Palatal Plate: An in-vitro study on flexural strength. Addit. Manuf..

[19] Lee S., Hong S.J., Paek J., Pae A., Kwon K.R., Noh K. (2019). Comparing accuracy of denture bases fabricated by injection molding, CAD/CAM milling, and rapid prototyping method. J. Adv. Prosthodont..

[20] Hsu C.Y., Yang T.C., Wang T.M., Lin L.D. (2020). Effects of fabrication techniques on denture base adaptation: An In Vitro study. J. Prosthet. Dent..

[21] Steinmassl O., Dumfahrt H., Grunert I., Steinmassl P.A. (2018). CAD/CAM produces dentures with improved fit. Clin. Oral Investig..

[22] Einarsdottir E.R., Geminiani A., Chochlidakis K., Feng C., Tsigarida A., Ercoli C. (2020). Dimensional stability of double-processed complete denture bases fabricated with compression molding, injection molding, and CAD-CAM subtraction milling. J. Prosthet. Dent..

[23] International Organization for Standardization (ISO) (1994). ISO 5725-1:1994 Accuracy (Trueness and Precision) of Measurement Methods and Results—Part 1: General Principles and Definitions.

[24] Hwang H.J., Lee S.J., Park E.J., Yoon H.I. (2019). Assessment of the trueness and tissue surface adaptation of CAD-CAM maxillary denture bases manufactured using digital light processing. J. Prosthet. Dent..

[25] Yoon H.I., Hwang H.J., Ohkubo C., Han J.S., Park E.J. (2018). Evaluation of the trueness and tissue surface adaptation of CAD-CAM mandibular denture bases manufactured using digital light processing. J. Prosthet. Dent..

[26] Emir F., Ayyildiz S. (2020). Accuracy evaluation of complete-arch models manufactured by three different 3D printing technologies: A three-dimensional analysis. J. Prosthodont. Res..

[27] VOCO GmbH (2019). V-Print Splint—Instructions of Use.

[28] Formlabs (2020). Dental LT Clear V1—Instructions of Use.

[29] Formlabs (2021). Dental Model V2—Instructions of Use.

[30] Dentaurum GmbH & Co. KG (2016). Orthocryl—Instructions of Use.

[31] 3Shape A/S (2017). 3Shape Dental System Catalog—Innovative 3D Scanning and CAD Solutions.

[32] Jeong Y.G., Lee W.S., Lee K.B. (2018). Accuracy evaluation of dental models manufactured by CAD/CAM milling method and 3D printing method. J. Adv. Prosthodont..

[33] Unkovskiy A., Schmidt F., Beuer F., Li P., Spintzyk S., Kraemer Fernandez P. (2021). Stereolithography vs. Direct Light Processing for Rapid Manufacturing of Complete Denture Bases: An In Vitro Accuracy Analysis. J. Clin. Med..

[34] Zhang Z.C., Li P.L., Chu F.T., Shen G. (2019). Influence of the three-dimensional printing technique and printing layer thickness on model accuracy. J. Orofac. Orthop..

[35] Xu Y., Xepapadeas A.B., Koos B., Geis-Gerstorfer J., Li P., Spintzyk S. (2021). Effect of post-rinsing time on the mechanical strength and cytotoxicity of a 3D printed orthodontic splint material. Dent. Mater..

[36] Ammoun R., Dalal N., Abdulmajeed A.A., Deeb G.R., Bencharit S. (2021). Effects of two postprocessing methods onto surface dimension of in-office fabricated stereolithographic implant surgical guides. J. Prosthodont..

[37] McLaughlin J.B., Ramos V., Dickinson D.P. (2019). Comparison of Fit of Dentures Fabricated by Traditional Techniques Versus CAD/CAM Technology. J. Prosthodont..

[38] Kim S.Y., Shin Y.S., Jung H.D., Hwang C.J., Baik H.S., Cha J.Y. (2018). Precision and trueness of dental models manufactured with different 3-dimensional printing techniques. Am. J. Orthod. Dentofac. Orthop..

[39] Osman R.B., Alharbi N., Wismeijer D. (2017). Build Angle: Does It Influence the Accuracy of 3D-Printed Dental Restorations Using Digital Light-Processing Technology?. Int. J. Prosthodont..

